# Vitamin B_12_ Supplementation: Is More Always Better?

**DOI:** 10.3390/nu18101597

**Published:** 2026-05-18

**Authors:** Manuela Yepes-Calderón, Caecilia S. E. Doorenbos, Mariken E. Stegmann, Daan J. Touw, Hermie J. M. Harmsen, M. Rebecca Heiner-Fokkema, Francjan J. van Spronsen, Eva Corpeleijn, Stephan J. L. Bakker

**Affiliations:** 1 Division of Nephrology, Department of Internal Medicine, University of Groningen, University Medical Center Groningen, 9713 GZ Groningen, The Netherlandss.j.l.bakker@umcg.nl (S.J.L.B.); 2 Department of Primary and Longterm Care, University of Groningen, University Medical Center Groningen, 9713 GZ Groningen, The Netherlands; 3 Department of Clinical Pharmacy and Pharmacology, University of Groningen, University Medical Center Groningen, 9713 GZ Groningen, The Netherlands; 4 Department of Microbiology, University of Groningen, University Medical Center Groningen, 9713 GZ Groningen, The Netherlands; 5 Department of Endocrinology and Metabolic Diseases, University of Groningen, University Medical Center Groningen, 9713 GZ Groningen, The Netherlands; 6 Department of Pediatric Metabolic Diseases, University of Groningen, University Medical Center Groningen, 9713 GZ Groningen, The Netherlands; 7 Department of Epidemiology, University of Groningen, University Medical Center Groningen, 9713 GZ Groningen, The Netherlands

**Keywords:** vitamin B_12_, cobalamin, supplementation, adverse effects

## Abstract

Vitamin B_12_ supplementation among people without proven deficiency has become popularized, driven by perceptions of (i) frequent underdiagnosis of deficiency, (ii) promotion as a natural enhancer of well-being, and (iii) a favourable safety profile. Here, we examine whether these claims align with current evidence. We present guidance from major health authorities, which advises against routine testing in asymptomatic individuals without risk factors. The prevalence of B_12_ deficiency varies greatly, mainly because definitions of B_12_ deficiency are not standardized and may include clinical, biochemical, or functional criteria. Biochemical deficiency (typically serum B_12_ < 148 pmol/L) is the predominant definition in epidemiological and clinical research studies. Using this criterion, deficiency appears uncommon in general populations of high-income countries (~2%), but substantially more frequent in settings with limited access to animal-source foods or B_12_-fortified products (up to 69%). Studying the effects of supplementation is also challenged by variation in the regimens used, which range from 0.02 to 1 mg/day orally and from 1 to 5 mg/week intramuscularly, with durations spanning ~4 weeks to ~7 years. This limits cross-study comparability. Overall, supplementation has not shown consistent benefits in populations without overt clinical or biochemical B_12_ deficiency, with no clear improvements in fatigue, mood, cognition, or cardiovascular outcomes. Benefits, when reported, appear confined to selected subgroups (e.g., hyperhomocysteinemia or low–normal B_12_ status). B_12_ supplementation is generally well tolerated. There are rare reports of acneiform and hypersensitivity responses, although these cannot be completely distinguished from reactions to, e.g., excipients. Observational studies associate B_12_ supplementation and higher circulating B_12_ levels with increased risks of malignancy. However, these findings are inconsistent, and current evidence is insufficient to establish causality, as potential reverse causation remains a major concern.

## 1. Introduction and Methods

Vitamin B_12_ supplements are usually prescribed to cover deficiencies and, less frequently, as cofactor therapy for rare inborn errors of metabolism [1,2]. In recent years, vitamin B_12_ supplementation has expanded to widespread over-the-counter use in people without proven deficiency, driven by perceptions of frequent underdiagnosis and by promotion as a natural enhancer of energy and well-being [3,4]. There is also the assumption that a water-soluble vitamin carries negligible risk [4,5]. A critical appraisal is warranted to delineate evidence-based indications, clarify benefits and harms in replete populations, and guide prudent dosing and monitoring.

We conducted a narrative review following the SANRA quality assessment recommendations. We aimed to evaluate vitamin B_12_ supplementation, with special interest in adults without established deficiency. For initial contextualization, we examined the recognized biological functions of vitamin B_12_, its main dietary sources, and the proposed reference intakes. To evaluate whether B_12_ is actually a “frequent deficiency”, we assessed how vitamin B_12_ deficiency is defined across the literature and the reported prevalence of deficiency in general populations. To investigate effects beyond deficiency correction, we examined evidence on vitamin B_12_ pharmacokinetics and routes of administration, as well as studies assessing potential health benefits in non-deficient individuals. We also reviewed reported adverse effects associated with supplementation and the potential mechanisms underlying these reactions.

To this end, we searched MEDLINE/PubMed, Embase, the Cochrane Library, and Web of Science (January 1990 to November 2025; English), and complemented database searches by screening authoritative sources, including guideline repositories (e.g., NICE and ADA) and informational resources (e.g., NIH Office of Dietary Supplements). Search strategies combined controlled vocabulary and free-text terms (the terms used are provided in Supplementary Table S1). Eligible evidence included randomized, cohort, case–control and cross-sectional studies; systematic reviews and meta-analyses, and authoritative guidelines. Single-case reports were excluded except when describing rare adverse events. Non-human studies were used only to support plausible pathophysiological mechanisms. Given substantial heterogeneity across populations, interventions, biomarkers, and outcomes, synthesis was qualitative, and findings are presented thematically.

Because assessment of variability in the definition of vitamin B_12_ deficiency was itself an objective of the literature search we performed, we did not restrict inclusion to a single diagnostic threshold or biomarker approach. To minimize ambiguity, whenever the term “vitamin B_12_ deficiency” is used, we report the specific criteria applied in the referenced source. We defined supplementation as administration of vitamin B_12_ via pharmacological formulations and applied no restrictions on dose, duration, or route. When supplementation regimens are discussed, we specify the dose and duration. For evidence regarding supplementation beyond deficiency correction, we prioritized studies that excluded individuals with overt deficiency or that evaluated supplementation effects in general or asymptomatic populations, and we report the principal inclusion criteria for each study cited.

This review has limitations inherent to its narrative design. Literature identification was not systematic, and no formal inclusion or exclusion criteria, study quality assessment, or risk-of-bias evaluation was performed. As a result, the synthesis may not capture all available evidence, and selective emphasis cannot be fully excluded.

## 2. Vitamin B_12_ Function and Sources

Vitamin B_12_ (cobalamin) is a vital water-soluble cofactor that supports methylation, DNA synthesis, myelin integrity, hematopoiesis, mitochondrial energy, and detoxification of propionate-derived carbon in the TCA cycle [6,7,8,9]. During pregnancy, increased B_12_ supports DNA synthesis, neurodevelopment, and red blood cell formation for maternal growth and fetal development [10].

Humans obtain vitamin B_12_ from external sources, i.e., animal-derived foods such as meat, fish, shellfish, eggs, and dairy products. Representative amounts include ~70 µg per 85 g of beef liver, ~20 µg per 85 g of pork liver, ~2.6 µg per 85 g of cooked salmon, ~2.4 µg per 85 g of cooked beef steak, ~1.3 µg per cup (240 mL) of milk, and ~0.5 µg per large egg [9,11,12]. Although trace amounts of vitamin B_12_ have been detected in certain plant-based foods, including marine algae, wild mushrooms, and brewer’s or nutritional yeast, these foods are not reliable sources of biologically active vitamin B_12_ [13]. Consequently, the only dependable plant-based sources of vitamin B_12_ are foods fortified with synthetic vitamin B_12_, such as certain cereals and vegetarian meat replacement products, which typically provide 0.5–1.8 µg per 100 g [12].

Recommended intakes vary by authority. In the United States, the Recommended Dietary Allowance (RDA) for adults is 2.4 µg/day. This recommended intake increases to 2.6 µg/day in pregnancy and 2.8 µg/day during lactation [9]. These values are derived from an Estimated Average Requirement intended to maintain hematologic status and normal serum B_12_. In Europe, the European Food Safety Authority (EFSA) suggests Adequate Intakes (AIs) of 4.0 µg/day for adults, 4.5 µg/day in pregnancy, and 5.0 µg/day during lactation, anchored to intake–biomarker relationships across multiple B_12_ status indicators in adults [14]. However, for pregnant and breastfeeding women, these recommendations were derived from assumptions regarding fetal vitamin B_12_ accumulation, breast-milk cobalamin concentrations, and average milk transfer [14]. Some national assessments have noted that, despite EFSA’s conservative approach, evidence suggests that lower intakes may be sufficient for many adults [15]. The Netherlands, for example, released in 2023 an RDA of 3.3 µg/day during pregnancy and 3.8 µg/day during lactation [16].

## 3. Is Vitamin B_12_ a Frequent Deficiency?

Assessing the prevalence of vitamin B_12_ deficiency remains challenging for several reasons. First, the absence of an international consensus on screening indications leads to substantial variability in the base populations across studies. Most guidelines agree on recommending against universal screening, as reflected in the advice of major health authorities, including the American Academy of Family Physicians (AAFP) in the United States [17], the National Institute for Health and Care Excellence (NICE) in the United Kingdom [3], The Dutch College of General Practitioners (NHG) in the Netherlands [18], and the British Columbia (BC) Provincial Guidelines in Canada [19]. However, the criteria for when to screen vary significantly. Some, such as the BC, recommend testing primarily based on the presence of risk factors such as low intake or diseases that reduce B_12_ absorption. Others, like the NHG [18], emphasize testing preferentially when abnormalities suggestive of B_12_ clinical deficiency are present, like unexplained neurological symptoms or macrocytosis/non-iron-deficiency anemia. Stricter guidelines, such as those from the AAFP and NICE [3,17], require the coexistence of both clinical features and at least one risk factor before recommending evaluation for vitamin B_12_ deficiency.

Second, the literature uses heterogeneous definitions of “deficiency”, including clinical deficiency (symptomatic disease attributable to B_12_ deficiency), biochemical deficiency (abnormal biomarkers in the absence of specific symptoms), and functional deficiency (biomarkers suggesting impaired B_12_ activity). In addition, no single biomarker is universally accepted as a gold standard for biochemical or functional deficiency, and laboratory cut-offs vary by assay and population (Table 1) [3,20]. Total serum B_12_ is the most widely available test, with common cut-offs of <148 pmol/L for deficiency. This marker is known to have limited sensitivity and specificity for functional deficiency. Values near the reference range are further complicated by assay imprecision (about 5–10% CV) and within-person variation (about 10–20%) [3,21]. For functional deficiency, markers such as methylmalonic acid (MMA) and total homocysteine (tHcy) better reflect intracellular cobalamin insufficiency, yet both rise with declining renal function. tHcy is also pre-analytically demanding, requiring rapid sample processing, and is influenced by folate, vitamin B_6_, and other metabolic factors [9,22]. Holotranscobalamin (holoTC), the transcobalamin-bound fraction that delivers B_12_ to cells, correlates slightly better with metabolic sufficiency than total B_12_ alone, but is less widely available [21]. Composite indices such as the combined indicator (cB_12_), which integrates total B_12_, holoTC, MMA, and tHcy, improve discrimination in the subclinical deficiency ranges, though they remain research tools rather than routine practice [23].

For these reasons, estimates of the frequency of vitamin B_12_ deficiency in the general population vary widely depending on both the population studied and the definition used (Table 1). Across the included studies, populations with adequate intake of animal-source foods, such as those in the United States and South Korea [24,25], consistently show low prevalence (generally <5%), whereas substantially higher rates are observed in settings where such foods are limited, as seen in India (~47% or higher) [26]. Even within high-income countries, important at-risk subgroups emerge; for example, women of childbearing age in the United Kingdom and Saudi Arabia show deficiency rates of around 6–12%, despite apparently adequate intake [27,28], suggesting potential gaps related to absorption, dietary quality, or reporting. Age is another consistent determinant, with older adults more likely to exhibit suboptimal status across populations [29]. In addition to true population differences, methodological factors substantially influence prevalence estimates. Depending on whether biochemical or functional markers are used, estimates within the same population can vary several-fold (e.g., approximately 2–3% vs. up to ~8% in U.S. data) [25], highlighting the impact of biomarker selection.

**Table 1:** Reported B12 deficiency prevalence among different populations.

Author	Publication Year	Year of Measurements	Country	Population	Definition Used	Deficiency Prevalence
Papakitsou et al. [29]	2024	~2020–2022	Greece	Older hospitalized adults	Serum B_12_ < 200 pg/mL	9%
Song et al. [24]	2023	2013–2015	South Korea	General population	Serum B_12_ < 148 pg/mL	3% (males) <2% (females)
Mineva et al. [25]	2021	1999–2004	USA	General population	cB12 Serum B_12_ < 148 pmol/L Serum tHcy > 13 µmol/L	3% (cB12); 2% (B12) 8% (tHcy)
Karakaş et al. [30]	2021	2018–2019	Turkey	Adolescents who visited the hospital	Serum B_12_ < 200pg/mL	~69%
Al-Musharaf et al. [28]	2020	~2015–2018	Saudi Arabia	Women of Childbearing Age	Serum B12 ≤ 220 pmol/L	~6%
Singla et al. [26]	2019	~mid-2010s	North India	General population	Serum B_12_ < 148 pmol/L	~47%
Sukumar et al. [27]	2016	~2008–2012	UK	Women of childbearing age	Serum B_12_ ≤ 150 pmol/L	12%

## 4. Routes and Forms of B_12_ Supplementation

Cyanocobalamin, hydroxocobalamin, and methylcobalamin are the principal crystalline forms of vitamin B_12_ used for supplementation. Delivery routes include oral and sublingual formulations, intramuscular or subcutaneous injections, and an intranasal cyanocobalamin spray for maintenance with amounts ranging from 100 to 3000 µg per dose [31]. The oral formulation, as B_12_ obtained from diet, relies primarily on intrinsic factor and saturates at roughly 1–2 µg per dose, beyond which a small passive-diffusion component accounts for additional uptake [4]. Sublingual dosing achieves similar biomarker responses to swallowed tablets, implying the same gastrointestinal pathway [32,33]. Following IM or SC injection, vitamin B_12_ is rapidly distributed (peaking at around 1 h), circulates bound to transcobalamin or haptocorrin, and is mainly stored in the liver with subsequent enterohepatic recirculation [9,34]. Intranasal cyanocobalamin is absorbed across the nasal mucosa, bypasses intrinsic factor, and is intended for maintenance after repletion [35].

For patients with confirmed clinical or biochemical deficiency, the choice of supplementation route is commonly guided by patient preference and cost [3,9,36,37]. A 2018 Cochrane systematic review [38], subsequent evidence summaries from the AAFP in 2022 [39], and a 2024 network meta-analysis [33] all found no clinically meaningful differences in efficacy among oral and parenteral routes, as all these routes reached normalization of the B_12_ values even in malabsorptive etiologies or severe clinical deficiency. Cost considerations generally favor high-dose oral cyanocobalamin as the lowest-cost option [40,41], given that it is self-administered. Because vitamin B_12_ supplementation is not routinely recommended for non-deficient individuals, there are no recommendations regarding the optimal route of administration in this population.

## 5. Supplementation Beyond Sufficiency: Are There Benefits?

Multiple randomized trials have examined whether vitamin B_12_ supplementation improves fatigue and general well-being in populations without overt B_12_ deficiency or B_12_-related anemia [42,43,44], including older adults, patients in secondary cardiovascular prevention, and individuals with elevated MMA. These studies have consistently shown no clinically meaningful benefit (Table 2). Notably, all of these studies enrolled selected clinical populations rather than generally healthy individuals, which limits the generalizability of their findings. This limitation was also highlighted in the systematic review by Markun et al., which assessed vitamin B_12_ supplementation in broader non-deficient populations but identified only a single eligible study [45]. Given the scarcity of eligible evidence the authors did not provide a quantitative meta-analytic estimate for fatigue outcomes.

For cognition and neurodegeneration, meta-analyses in participants without cognitive impairment or diagnosed clinical B_12_ deficiency (with variable definitions), covering a wide range of doses and durations, conclude that B-vitamin supplementation does not meaningfully alter global cognitive trajectories in unselected populations [46,47] (Table 2). In contrast, the VITACOG trial in patients with mild cognitive impairment (MCI) who were not using B_12_ supplements at baseline (mean plasma B_12_ ~330 pmol/L) showed that combined supplementation with vitamin B_12_, folic acid, and vitamin B_6_ for 24 months slowed brain atrophy and cognitive decline, particularly among participants with elevated homocysteine or sufficient long-chain omega-3 status [48,49]. The authors proposed several mechanisms to explain this interaction, including that homocysteine lowering may restore pathways involved in the incorporation of docosahexaenoic acid (DHA) into neuronal membranes, making structural benefits more evident when omega-3 substrate availability is adequate. In addition, both omega-3 fatty acids and B vitamins may reduce tau hyperphosphorylation and neurofibrillary tangle formation, and both nutrient classes may attenuate neuroinflammation [50]. However, because these effects derive from a single study and were achieved using combined B-vitamin supplementation, vitamin B_12_-specific effects cannot be isolated or confirmed.

Regarding mood, two pooled analyses of randomized trials in individuals without depression at baseline show no preventive effect of vitamin B_12_ supplementation on depressive symptoms [46,51]. Similarly, in individuals with existing depression, meta-analytic evidence indicates no consistent improvement in depressive symptoms with B-vitamin supplementation on top of regular antidepressant therapy compared to antidepressant therapy alone [52]. This is consistent with an RCT in adults ≥50 years with major depression, which found no difference in remission rates when B-vitamin supplementation (including 0.5 mg B_12_) was added to antidepressant therapy [53]. In contrast, a randomized trial in patients with depression and low–normal vitamin B_12_ levels (190–300 pg/mL) reported higher response rates when injectable B_12_ was added to standard antidepressant treatment, suggesting a potential benefit in this subgroup (Table 2).

Finally, in cardiovascular prevention, only one meta-analysis assessed effects in the general population, with a focus on regions without mandatory folate fortification [54]. This study reported a modest reduction in stroke risk with folic acid-based regimens combined with vitamin B_12_; however, the effect was not significant in analyses restricted to trials using higher B_12_ doses (>4 mg/day). This may reflect reduced statistical power due to smaller sample sizes, but also suggests that the observed benefit is likely driven primarily by folate rather than vitamin B_12_. Most of the remaining evidence comes from secondary prevention trials, including large randomized studies such as NORVIT, VITATOPS, HOPE-2, and SU.FOL.OM3, conducted in patients with recent cardiovascular events or established cardiovascular disease, which showed no reduction in major adverse cardiovascular events with multiple B-vitamin supplementation, including B_12_ [55,56,57,58] Notably, HOPE-2 reported a 25% relative reduction in stroke despite null effects on the composite endpoint (cardiovascular death, myocardial infarction, and stroke) [58]. This finding is consistent with the meta-analytic evidence; however, the specific contribution of vitamin B_12_ cannot be isolated, as all interventions involved combined B-vitamin regimens.

**Table 2:** Studies regarding potential benefits of B_12_ supplementation in nondeficient populations.

Author	Year	Study Type	Population Characteristics	*n*	B_12_ Treatment	Comparator	Outcome	Effect (95% CI or *p* Value)	**Subgroups**
**Fatigue and well-being**									
Dangour et al. [43]	2015	RCT	≥75 years and moderate vitamin B_12_ deficiency (107–210 pmol/L) without anemia	201	1 mg B_12_ daily for 12 months	Placebo	30-item General Health Questionnaire score	SMD −0.1 (−1.2, 1.0)	—
Andreeva et al. [44]	2014	RCT	Survivors of stroke, myocardial infarction, or unstable angina	2501	Multivitamins with 0.02 mg B_12_ daily for a median of 4.7 years	Placebo	SF-36	SMD 0.9 (−0.5, 2.4)	—
Hvas et al. [42]	2003	RCT	Adults with elevated MMA (0.4–2.0 μmol/L^−1^)	140	1 mg B_12_ injection weekly for 4 weeks	Placebo	SF-36	SMD 0.7(−3.7, 5.0)	—
**Cognition**									
Markun et al. [46]	2021	Meta-analysis	Patients without advanced neurological disorders or overt B_12_ deficiency	6276	Between 0.1 mg to 1 mg daily orally or IM. Mean treatment duration from 4 to 117 week	Placebo or other vitamin complex	Cognitive executive function (different scales)	SMD 0.06 (−0.02, 0.14)	—
Behrens et al. [47]	2020	Meta-analysis	Cognitively unimpaired individuals	12,697	Between 0.1 mg to 1 mg daily orally or IM. Mean treatment duration from 6 months to 7 years	Placebo or other vitamin complex	Global cognition (different scales)	SMD 0.02 (−0.034, 0.08)	—
Oulhaj et al. [49].	2016	RCT (VITACOG)	≥70 years with MCI	266	B_12_ 0.5 mg + Folic acid 0.8 mg + B6 20 mg daily for 24 months	Placebo	TICS-M	Intervention 24.8 vs. placebo 24.9 (*p* = 0.66)	Treatment effect difference between the highest and lowest omega-3 tertiles 2.85 points (*p* = 0.035).
Smith et al. [48]	2010	RCT (VITACOG)	≥70 years with MCI	168	B_12_ 0.5 mg + Folic acid 0.8 mg + B6 20 mg daily for 24 months	Placebo	Mean rate of brain atrophy	Intervention 0.76% vs. placebo 1.08% (*p* = 0.001)	The rate of atrophy in patients with homocysteine > 13 µmol/L was 53% lower in the active treatment group vs. placebo (*p* = 0.001).
**Mood/Depression**									
Markun et al. [46].	2021	Meta-analysis	Patients without advanced neurological disorders or overt vitamin B_12_ deficiency	6276	Between 0.1 mg to 1 mg daily orally or IM. Mean treatment duration from 4 to 117 week	Placebo or other vitamin complex	Depressive symptoms (different scales)	SMD −0.05 (−0.15, 0.05)	—
Almeida et al. [51]	2015	Meta-analysis	Adults without and without depressive episode at the time of randomization	1242 (without) 505 (with)	Between 0.1 to 1 mg alone or in combination with other vitamins or with antidepressants (for depressive episode). Mean treatment duration from 4 weeks to 7 years	Placebo or antidepressant alone	Depressive symptoms (different scales)	Without depressive episode SMD −0.05 (−0.16, 0.06) With depressive episode SMD −0.12 (−0.45, 0.22)	—
Almeida et al. [52]	2014	RCT	≥50 years with major depressive episode	153	Citalopram together with B_12_ 0.5 mg + folic acid 2 mg + B6 25 mg for 52 weeks	Citalopram only	Remission of the depressive episode	78.1 intervention vs. 79.4% control (*p* = 0.84)	—
Syed et al. [53].	2013	RCT	Depressive episode and low–normal B_12_ (190 and 300 pg/mL)	199	B_12_ 1 mg IM weekly + antidepressants for 6 weeks	Only antidepressants	20% Reduction in the HAM-D score	100% intervention vs. 69% control (*p* < 0.001).	—
**Cardiovascular prevention**									
Hsu et al. [54].	2018	Meta-analysis	General population of areas without mandatory folate fortification	65,812	B_12_ (0.02–1 mg) + folic acid (0.4–2.5) mg for 2–7 years	Placebo/usual care	Stroke	RR 0.85 (0.77–0.95)	Folic acid + ≥0.4 mg/day B_12_ RR 0.95 (0.86, 1.05)
Galan et al. [55].	2010	RCT (SU.FOL.OM3)	Patients with prior MI, stroke or angina	2501	B_12_ 0.02 mg + folic acid 0.56 mg + B_6_ 3 mg daily for median 4.7 years	Placebo	Stroke, MI, or vascular death	HR 1.01 (0.81–1.26)	
Hankey et al. [56]	2010	RCT (VITATOPS)	Patients with recent stroke or TIA	8164	B_12_ 0.5 mg + folic acid 2 mg + B_6_ 25 mg daily for median 3.4 years	Placebo	Stroke, MI, or vascular death	RR 0.91 (0.82–1.00)	
Bønaa et al. [57]	2006	RCT (NORVIT)	Patients with recent MI	3749	B_12_ 0.4 mg + folic acid 0.8 mg + B_6_ 40 or B_12_ 0.4 mg + folic acid 0.8 mg for median of 40 months.	Placebo	Recurrent MI, stroke, or sudden death	Vitamin B_12_ + folic acid RR 1.08 (0.93, 1.25). Vitamin B_12_ + folic acid + vitamin B_6_ RR 1.22 (1.00, 1.50).	
Lonn et al. [58]	2006	RCT (HOPE-2)	≥55 years with previous vascular disease or diabetes	5522	B_12_ 1 mg + folic acid 2.5 mg + B_6_ 50 mg daily for 5 years	Placebo	Stroke, MI, or vascular death	RR 0.95 (0.84, 1.07)	For stroke RR 0.75 (0.59, 0.97)

CI, confidence interval; HAM-D, Hamilton Rating Scale for Depression (Urdu version); HR, hazard ratio; IM, intramuscular; MCI, mild cognitive impairment; MI, myocardial infarction; RCT, randomized clinical trial; RR, relative risk; SMD, standardized mean difference; TIA, transient ischemic attack; TICS-M, Telephone Interview for Cognitive Status–modified.

## 6. Supplementation: Are There Potential Harms?

Vitamin B_12_ has low acute toxicity, and no tolerable upper intake level has been established [9,59]. The supplementation doses mentioned in the studies above, as well as the much higher doses used in, e.g., the treatment of inborn errors of metabolism, are generally well tolerated [1,2]. Nevertheless, rare adverse reactions have been reported, and observational and experimental studies have raised questions about the possible long-term effects of high B_12_ exposure. However, most evidence is of low quality and remains hypothesis-generating (Table 3).

Dermatologic reactions have been reported in multiple case reports linking acneiform eruptions and rosacea flares to vitamin B_12_ supplementation, either as high-dose injections or combined oral preparations. These reactions typically resolved after discontinuation of the supplement [60,61,62,63] (Table 3). However, B_12_-specific effects are difficult to isolate because these products are often administered as multivitamin formulations, and reactions may also be attributable to other vitamins, excipients, or preservatives. A proposed mechanism, supported by human microbiome studies, is that exogenous vitamin B_12_ uptake by *Cutibacterium acnes* downregulates cobalamin biosynthesis pathways and increases porphyrin production, a pro-inflammatory signal, potentially due to a shift in metabolic allocation away from de novo B_12_ synthesis [64].

Hypersensitivity reactions to vitamin B12 are reported, but they are rare and mainly immediate, including occasional anaphylaxis after high-dose parenteral cyanocobalamin or hydroxocobalamin (1–5 mg) [65,66,67]. In a 29-patient series, most cases were immediate reactions (62%), including anaphylaxis, with some later linked to excipients such as PEG. In the same study, delayed reactions were less frequent and often still compatible with tolerance of alternative oral or injectable formulations [68]. Case reports similarly show that oral vitamin B12 is frequently tolerated after parenteral reactions, supporting a possible role for formulation components rather than cobalamin itself in some cases [65,67]. Cobalt allergy is also relevant, as vitamin B12 contains cobalt at its core, and a subset of referred patients are ultimately sensitized to cobalt rather than the full vitamin B12 preparation [68,69].

**Table 3:** Reported potential adverse effects of B12 supplementation.

Author	Publication Year	Study Type	Population	*n*	B_12_ Treatment	Reaction/Effect Reported
**Dermatological reactions**						
Feng et al. [60]	2025	Case report	Adult with rosacea	1	Daily compound vitamin tablets which included B_12_ −4.0 µg	Gradually worsening erythematous papules on the face, accompanied by itching, burning, stinging, and skin tightness. Resolved with discontinuation
Bahbouhi et al. [61]	2023	Case report	Adult with pernicious anemia	1	Weekly IM hydroxocobalamin 5000 μg	A sudden, extensive, and monomorphic eruption of inflammatory papulo-pustules and nodules, affecting the face and the trunk. Resolved with discontinuation and treatment with Lymecycline.
Bowden et al. [62]	2023	Case report	Adult patient	1	Over-the-counter vitamin B_12_ weekly (exact composition none described)	Monomorphic erythematous papules and pustules on the face, chest, arms, and back. Resolved with discontinuation and a course of doxycycline.
Jansen et al. [63]	2001	Case report	17 years old patient	1	B_12_ 20 mcg + B6 80 mcg	Rosacea fulminans. Improved after discontinuation and treatment with methylprednisolone and isotretinoin
**Hypersensitivity reactions**						
Ullah et al. [65]	2018	Case report	Adult patient with megaloblastic anemia	1	1 mg cyanocobalamin IM	Anaphylactic reaction Resolved with emergency treatment. Patient later tolerated oral vitamin B_12_ without side effects
El Rhermoul et al. [68]	2024	Retrospective multicenter study	Patients referred with the diagnosis “Vit B12 hypersensitivity”	29	Skin prick testing (1 mg/mL) with cyanocobalamin and hydroxocobalamin and intradermal testing. If negative skin tests, Vit B_12_ DPT was done with either the index or an alternative drug.	18 (62%) had immediate Vit B12 hypersensitivity: 8 anaphylaxes (7 to IM of which 1 PEG-related). Some tolerated alternative B_12_ 8 delayed reactions: some tolerated alternative IM or oral formulations; 3 were referred for cobalt allergy.
Branco-Ferreira et al. [66].	1997	Case report	Adult patient	1	Hydroxocobalamin 5000 mcg	Anaphylactic reaction that resolved with acute treatment. Patient underwent a desensitization process
Bilwani et al. [67]	2005		Adult patient with megaloblastic anemia	1	1 mg cyanocobalamin intramuscularly	Resolved with emergency treatment (epinephrine/supportive care). On a later visit, she was offered a high oral dose (2 mg/day), which was tolerated without any unwanted side effects
**Malignancy**						
Brasky et al. [70].	2017	Cohort	Adults (VITAL cohort)	77,118	Self-reported B_12_ ≥ 55 µg/day long-term supplementation	Lung cancer among men HR 1.98 (95% CI 1.32, 2.97). No effect in women.
Ebbing et al. [71].	2009	RCT	Patients with ischemic heart disease	6837	B_12_ 0.4 mg + Folic acid 0.8 mg ± B_6_ 0.4 mg daily for a median of 40 months	All-cancer incidence HR 1.21 (95% CI 1.03, 1.41) and cancer-related mortality HR 1.38 (1.07, 1.79).
Zhang et al. [72].	2008	RCT	Female > 42 years with preexisting cardiovascular disease	5442	B_12_ 1 mg + Folic acid 2.5 mg + B_6_ 50 mg	Invasive cancer incidence HR 0.97 (95% CI 0.79, 1.18)

CI, confidence interval; DPT, drug provocation test; HR, hazard ratio; IM, intramuscular; RCT, randomized clinical trial.

Beyond acute adverse effects, long-term supplementation with vitamin B_12_ has also been linked to other potential risks, particularly cancer. This association remains debated. In a Norwegian randomized trial on patients with ischemic heart disease, participants receiving vitamin B_12_ + folic acid for approximately 3 years had a higher cancer incidence (10%) than controls (8.4%; *p* = 0.02), although the specific contribution of vitamin B_12_ versus folic acid cannot be disentangled [71]. In contrast, another randomized trial in U.S. women using combined B vitamins, including vitamin B_12_ (1 mg/day) for about 7 years, did not replicate this finding [72]. Observational evidence from the VITAL cohort reported an increased lung cancer risk in men using long-term vitamin B_12_ supplements (self-reported > 55 µg/day based on a 10-year average), independent of multivitamin use [70], but these results have not been replicated by other studies. Much of the remaining evidence does not assess supplementation directly but rather circulating vitamin B_12_ levels. Three cohort studies on the adult general population (sample sizes between 688 and 757K participants) examined the association of elevated plasma B_12_—either as a continuous variable or using thresholds (e.g., ≥1000 ng/L)—with incident cancer within relatively short follow-up periods (1–12 months). These studies reported increased cancer risk, with effect estimates ranging from an OR of 1.15 (95% CI 1.06, 1.25) per standard deviation increase to an HR of 5.9 (95% CI 2.79,12.45) for markedly elevated levels (≥1000 ng/L) [73,74,75]. Given the observational nature of these studies, causality remains uncertain. The short interval between B_12_ measurement and cancer diagnosis suggests that elevated B_12_ may reflect reverse causation or disease-related alterations in B_12_ metabolism rather than a direct carcinogenic effect or the effect of external B12 exposure. Consistent with this interpretation, Mendelian randomization analyses examining genetically predicted B_12_ levels have not demonstrated a causal relationship with cancer risk [76,77].

Other concerns have been raised about potential adverse effects of sustained high vitamin B_12_ levels. Observational studies in the general population, including the PREVEND cohort (the Netherlands), have reported associations between higher circulating B_12_ concentrations and increased mortality risk (HR 1.25; 95% CI 1.06–1.47 per 1-SD increase) [78]. Meta-analytic evidence from 22 cohort studies similarly shows a modest, linear increase in mortality per 100 pmol/L rise in serum B_12_ (HR 1.04; 95% CI 1.01–1.08), with stronger associations in older adults and at higher concentrations (≥600 pmol/L: HR 1.50; 95% CI 1.29–1.74) [79]. However, it is important to emphasize that these findings relate to circulating B_12_ levels rather than supplementation per se. Interpretation is further limited by the observational design of these studies, where reverse causation is highly plausible. Elevated B_12_ is frequently observed in conditions such as liver disease, renal dysfunction, malignancy, and systemic inflammation, all of which are independently associated with increased mortality [79,80,81]. Experimental studies also suggest that high exogenous B_12_ availability may influence microbial metabolism and growth, potentially promoting species such as *Salmonella enterica* and *Listeria monocytogenes* [82,83]. While this provides biological plausibility for downstream effects, including infection risk or metabolic byproducts, the clinical relevance in humans remains uncertain.

## 7. Knowledge Gaps

**Figure 1:**
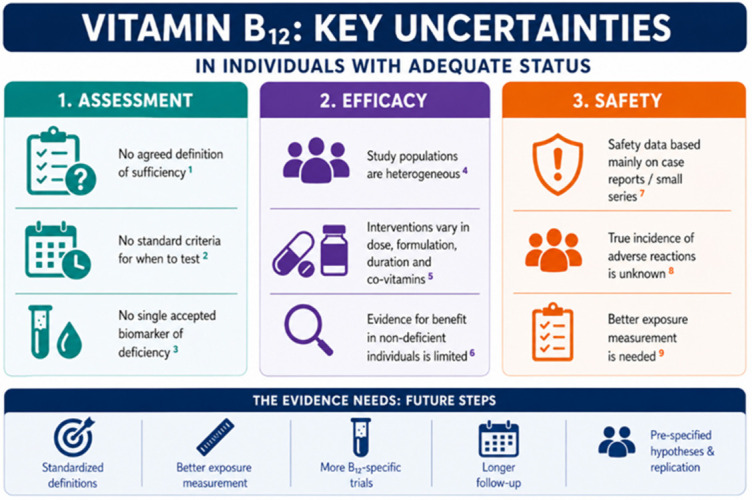
Knowledge gaps regarding B12 supplementation in individuals with adequate status. This figure summarizes the main gaps identified in the different sections of the manuscripts, for which the following sources are cited: ^1^ [3,20], ^2^ [3,17,18,19], ^3^ [3,9,21,22,23], ^4,5^ [42,43,44,46,47,51,55,56,57,58], ^6^ [42,43,44,46,47], ^7,8^ [60,61,62,63,65,66,67], ^9^ [73,74,75,79].

Key uncertainties remain regarding the assessment, efficacy, and safety of vitamin B_12_ in individuals with adequate status (Figure 1).

For assessment, the main limitation is the absence of a universally accepted definition of “true” vitamin B_12_ sufficiency or insufficiency. The current guideline approaches identified in this Review often favor a clinical definition that incorporates symptoms and risk factors to guide testing, but there is still no consensus on which specific criteria should be applied. Likewise, there is no single accepted biochemical marker of deficiency. Plasma B_12_ is the most commonly used measure because it is widely available, but it has recognized limitations and only a modest relationship with functional deficiency. Other biomarkers, including holotranscobalamin, methylmalonic acid, and homocysteine, each have important drawbacks that have limited their application in clinical practice and their homogeneous use. As a result, estimates of the prevalence of deficiency remain difficult to compare across studies, and agreement on a common operational definition is a necessary first step before the global burden of B_12_ deficiency can be meaningfully quantified.

The evidence for benefit of B_12_ supplementation in non-deficient populations is also limited by heterogeneity in the populations and interventions studied. Although several large trials are available, most tested combined multivitamin regimens rather than B_12_ alone, with substantial variability in dose, formulation, duration, and the type and concentration of accompanying vitamins. This makes pooling difficult and prevents clear attribution of effects specifically to vitamin B_12_. Most trials were negative overall, but definitive conclusions about B_12_ itself remain limited because the independent contribution of the vitamin has rarely been isolated. In addition, many subgroup findings appear to come from post hoc analyses rather than pre-specified hypotheses, and these results have not been consistently replicated. External validation is therefore needed, particularly for signals suggesting benefit in specific contexts such as hyperhomocysteinemia or adequate omega-3 status.

Safety data also require clarification. Most reported adverse reactions are based on case reports or small case series, so the true incidence of dermatologic and hypersensitivity reactions following high-dose vitamin B_12_ remains poorly quantified. Given the apparent rarity of these reactions, larger observational studies are needed that address the limitations identified in the studies included in this review, particularly when supplementation is self-reported or when exposure is inferred from plasma B_12_ concentrations rather than documented supplement use. This distinction is critical, as elevated circulating B_12_ may reflect underlying disease, reverse causation, or metabolic dysregulation rather than supplementation itself. Future studies should therefore measure exposure more precisely, including the exact B_12_ formulation, dose, duration, route of administration, and the presence of excipients or co-administered vitamins, all of which may confound safety signals. In particular, studies examining high plasma B_12_ levels should be replicated using detailed supplementation data and long-term follow-up to distinguish true adverse effects of vitamin B_12_ from markers of underlying illness.

Overall, the evidence base would benefit from standardized definitions, better exposure measurement, more B_12_-specific trials, and longer follow-up. Until then, conclusions about the effects and safety of vitamin B_12_ beyond deficiency correction should be considered provisional.

## 8. Conclusions

Vitamin B_12_ is an essential nutrient, fundamental for one-carbon metabolism and mitochondrial energy production [6,7,8,9]; however, its evaluation, epidemiology, and management in contemporary populations remain complex.

Regarding the perception that vitamin B_12_ deficiency is “frequently underdiagnosed,” the reported prevalence in the general population varies widely (approximately 2–69%) [24], but appears consistently low in fortified, high-income settings [24,25] while remaining substantial in regions with limited access to animal-source foods or fortified alternatives, or where malabsorption is common [26,30]. However, the absence of consensus on screening indications and the wide variability in definitions limit comparability across studies.

Concerning the notion that vitamin B_12_ acts as a “natural well-being enhancer,” the evidence identified in this review does not support routine supplementation beyond sufficiency. Apart from correcting deficiencies or treating inherited metabolic disorders, supplementation has not shown consistent benefits for fatigue [42,43,44], mood [46,51], or global cognition [46,47]. Observed modest effects, particularly in cognitive or cardiovascular outcomes, appear limited to small subgroups (e.g., individuals with hyperhomocysteinemia or adequate omega-3 status [48,49]) rather than being generalizable. Interpretation is further complicated by substantial heterogeneity in study design, including wide ranges in dosing (0.02–1 mg) and duration (4 weeks to ~7 years) and frequent co-supplementation with other vitamins, which limits attribution of effects specifically to vitamin B_12_.

Regarding safety, vitamin B_12_ is generally well tolerated, even at doses exceeding recommended intakes. Rare dermatological (e.g., acneiform eruptions, rosacea flares) and hypersensitivity reactions have been reported, mostly in case studies, and typically resolve after discontinuation; however, these reports are limited in quality, and causality cannot be firmly established, as reactions may also be related to other vitamins, excipients, or preservatives [60,61,62,63,65,66,67]. One randomized controlled trial reported an increased cancer risk with combined B-vitamin supplementation including vitamin B_12_ [71]; however, this finding was not replicated in a later trial [72]. Observational studies have also reported associations between long-term high-dose supplementation and certain cancers (e.g., lung cancer in men) [73,74,75], but these findings are inconsistent [76,77], and their observational nature makes confounding a significant concern.

Finally, the studies presented in this review highlight several key gaps: the need for standardized definitions of vitamin B_12_ status, trials specifically evaluating vitamin B_12_ supplementation independent of other nutrients, and well-designed studies with precise exposure assessment and long-term follow-up to evaluate adverse effects. Addressing these limitations is essential to clarify whether the observed associations reflect true causal effects or are driven by underlying confounding factors.

These conclusions should be interpreted in light of the limitations inherent to a narrative review, including potential omissions in the literature, and should be considered descriptive and hypothesis-generating rather than definitive guidance for clinical decision-making.

## Data Availability

No new data were created or analyzed in this study. Data sharing is not applicable to this article.

## Supplementary Materials

The following supporting information can be downloaded at: https://www.mdpi.com/article/10.3390/nu18101597/s1, Table S1: Representative core search terms used for literature identification.

## Abbreviations

The following abbreviations are used in this manuscript:

AAFP	American Academy of Family Physicians
ADA	American Diabetes Association
AI	Adequate Intake
BC	British Columbia (provincial guideline)
cB_12_	Combined indicator of vitamin B_12_ status
CI	Confidence interval
DHA	Docosahexaenoic acid
EAR	Estimated Average Requirement
EFSA	European Food Safety Authority
holoTC	Holotranscobalamin
HR	Hazard ratio
IF	Intrinsic factor
IM	Intramuscular
IV	Intravenous
KNHANES	Korea National Health and Nutrition Examination Survey
MCI	Mild cognitive impairment
MMA	Methylmalonic acid
NHANES	National Health and Nutrition Examination Survey
NHG	Nederlands Huisartsen Genootschap (Dutch College of General Practitioners)
NICE	National Institute for Health and Care Excellence
PPI	Proton-pump inhibitor
RCT	Randomized controlled trial
RDA	Recommended Dietary Allowance
RNI	Reference Nutrient Intake
SC	Subcutaneous
tHcy	Total homocysteine
