# Supplementary material for: Vitamin B12 Supplementation: Is More Always Better?
Source: Nutrients. 2026 May 18;18(10):1597. doi: 10.3390/nu18101597 (PMC13209201; doi:10.3390/nu18101597)
Supplement: Supplementary file 1 [file nutrients-18-01597-s001.zip › nutrients-4057027-supplementary.pdf]

nutrients

Supplemental Material

Vitamin B_{12} supplementation: is more always better?

Manuela Yepes-Calderón [1,*], Caecilia S.E. Doorenbos [1], Mariken E. Stegmann [2], Daan J. Touw [3], Hermie J.M. Harmsen [4], M. Rebecca Heiner-Fokkema [5], Francjan J. van Spronsen [6], Eva Corpeleijn [7] and Stephan J. L. Bakker [1]

[1] Division of Nephrology, Department of Internal Medicine, University of Groningen, University Medical Center Groningen, Groningen, the Netherlands
[2] Department of Primary and Longterm Care, University of Groningen, University Medical Center Groningen, Groningen, the Netherlands
[3] Department of Clinical Pharmacy and Pharmacology, University of Groningen, University Medical Center Groningen, Groningen, the Netherlands
[4] Department of Microbiology, University of Groningen, University Medical Center Groningen, Groningen, the Netherlands
[5] Department of Endocrinology and Metabolic Diseases, University of Groningen, University Medical Center Groningen, Groningen, the Netherlands
[6] Department of Pediatric Metabolic Diseases, University of Groningen, University Medical Center Groningen, Groningen, the Netherlands
[7] Department of Epidemiology, University of Groningen, University Medical Center Groningen, Groningen, the Netherlands
* Correspondence: m.yepes.calderon@umcg.nl

Academic Editor: Firstname Lastname

Received: date
Revised: date
Accepted: date
Published: date

Citation: To be added by editorial staff during production.

Copyright: © 2025 by the authors. Submitted for possible open access publication under the terms and conditions of the Creative Commons Attribution (CC BY) license (https://creativecommons.org/licenses/by/4.0/).

Supplementary Table 1. Representative core search terms used for literature identification

Conceptual domain	Representative search terms
Population	"general population", "healthy adults", "asymptomatic adults", "non-deficient", "B12 sufficient", "community-dwelling"
Vitamin B_{12} / cobalamin	"vitamin B12", "cobalamin", "circulating B12", "serum B12", "plasma B12"
Biomarkers	"holotranscobalamin", "holoTC", "methylmalonic acid", "MMA", "homocysteine", "total homocysteine"
Deficiency / status	"vitamin B12 status", "B12 deficiency", "subclinical deficiency", "B12 sufficiency"
Supplementation	"vitamin B12 supplementation", "B12 supplements", "cyanocobalamin", "hydroxocobalamin", "methylcobalamin"
Pharmacokinetics/ absorption	"absorption", "bioavailability", "pharmacokinetics", "intestinal absorption"
Cardiovascular outcomes	"cardiovascular disease", "cardiovascular risk", "homocysteine", "cardiovascular outcomes"
Neurocognitive outcomes	"cognition", "neurocognitive function", "neurological outcomes", "cognitive decline"
Dermatologic events	"acneiform", "rosacea", "dermatologic adverse events"
Hypersensitivity	"hypersensitivity", "allergic reaction", "anaphylaxis"
Microbiome	"gut microbiome", "intestinal microbiota"

Search terms were used iteratively and in combination, employing both controlled vocabulary and free-text terms, and adapted across databases.
